# Effectiveness of community-based interventions for patients with schizophrenia spectrum disorders: a study protocol for a systematic review

**DOI:** 10.1186/s13643-021-01662-0

**Published:** 2021-04-13

**Authors:** Soo-Yeon Kim, Ah. Rim Kim

**Affiliations:** 1 Department of Nursing, Daegu Haany University, Hanuidae-ro, Gyeongsan-si, Gyeongsangbuk-do 38609 Republic of Korea; 2 Department of Nursing, Far East University, Chungchungbuk-do, South Korea

**Keywords:** Systematic review, Schizophrenia, Quality of life, Community mental health services, Study protocol

## Abstract

**Background:**

Schizophrenia requires a community-based intervention approach combined with standard treatment to prevent relapses. A literature review is required to understand the effectiveness of community-based interventions and to enhance quality in countries where they have not been fully established. This is a protocol for a systematic review of the effectiveness of community-based interventions for patients with schizophrenia spectrum disorders.

**Methods:**

We will search (from inception to January 2021) PubMed/MEDLINE, EMBASE, PsycINFO, CENTRAL, CINAHL, and Research Information Sharing Service/Korean databases. Randomized controlled trials on community-based interventions for patients with schizophrenia spectrum disorders will be eligible. The comparison groups will include patients with schizophrenia spectrum disorders who are only receiving the usual care and those who also receive community-based interventions. The schizophrenia spectrum disorders referred to in this study are defined according to the DSM-5: delusional disorders, schizophrenic disorders, and schizoaffective disorder will be included. Relapse/re-hospitalization rates (primary outcome) and quality of life (secondary outcome) will be identified for each group. Two reviewers will independently screen study titles, abstract data, and full-text articles and perform the data extraction process. Potential conflicts will be resolved through discussion. The study risk of bias will be appraised using the Cochrane Risk of Bias 2.0 tool. Results will be descriptively synthesized and will be structured according to patients’ characteristics, intervention type and exposure, and outcome type. If feasible and appropriate, outcome data will be used to perform random effects meta-analyses. Discrete variables will be calculated via odds ratio, and continuous variables will be calculated via standardized mean difference using RevMan 5.3 software.

**Discussion:**

We will provide a summary of the available evidence on the effectiveness of community-based interventions and specific guidelines to improve their outcomes.

**Systematic review registration:**

PROSPERO (CRD42019145660).

**Supplementary Information:**

The online version contains supplementary material available at 10.1186/s13643-021-01662-0.

## Background

Recently, in Korea, as violent crimes performed by patients with schizophrenia began to rise and became a social issue, the social atmosphere created a negative bias related to this mental illness [1]. Social issues related to patients with schizophrenia should be approached in a way that allows for the identification of blind spots in mental illness management, rather than just focusing on the crimes that occurred, and should also recognize the need for a closer look and care at the community level, which may ultimately help these individuals, when combined with regular treatment.

Schizophrenic episodes recur in patients at a high rate after diagnosis [2]. Relapse rates for schizophrenia are associated with the discontinuation of their respective antipsychotic drug treatments; therefore, symptoms such as violence are relatively well controlled―unlike social prejudice―when drug treatment is well adapted to the patient’s life [3]. Contrastingly, untreated schizophrenia tends to lead patients to repeated hospitalization due to frequent symptomatic relapses, a process that eventually leads to a general deterioration in individuals’ quality of life, provoked by difficulties related to cognitive skills, communication and interpersonal relationships, and significant social withdrawal [4–6].

Additionally, schizophrenia has been shown to have a higher requirement related to family care compared to other chronic conditions [7], mainly because it is difficult to maintain patients’ insight into their treatment and drug compliance, as well as manage their symptoms, which is represented by high relapse rates. Thus, schizophrenia requires long-term comprehensive care combined with standard treatment, to prevent recurrence and improve the individuals’ function on a daily basis.

Several studies have shown positive effects of interventions when used in parallel with the standard treatment for the management of schizophrenia. In groups who present low drug compliance and violent tendencies, symptoms were significantly reduced after more than 6 months of assisted outpatient treatment [8]. The results of a 2018 meta-analysis suggested that community-based interventions that are performed in the initial stages of the mental illness are effective to diminish the symptoms of schizophrenia, compared to the standard treatment [9]. Additionally, case management for more than 2 years has shortened the length of re-hospitalization [10].

Community-based interventions have shown to be effective not only in terms of the costs associated with hospitalization but also in terms of the quality of life and family burden of patients [11]. Particularly, it has been shown to help patients with schizophrenia in maintaining education and/or getting a job, which has resulted in their social reintegration and personal development [12].

Based on these positive effects of community involvement, many countries encourage community-based interventions. The National Institute of Clinical Excellence Guidelines in the UK emphasizes the need for community-based psychiatric intervention, such as cognitive therapy, counseling, and family intervention, in addition to standard treatment [13]. In Korea, since the enactment of the Mental Health Act in 1995, a policy of deinstitutionalization has been established with the help of the Mental Health Center, and community-based case management programs for various mental disorders have been developed and carried out [11, 14]. However, to date, they have not been fully established, and community support systems are still vulnerable [15].

Thus, we deem that a systematic review on community-based intervention programs related to the treatment of schizophrenia is required. Similar studies were conducted in 2014 [16] and 2017 [17]; however, those studies were limited to low- and middle-income countries and they cannot be considered an international standard, thereby limiting the usefulness of their findings. The community-based interventions for schizophrenia spectrum patients were reviewed comprehensively in 2013 [18], but the effectiveness of the community program was not known because meta-analysis was not carried out.

This study protocol is not limited to a specific country or economic level, but plans an overall review, allowing the results to be presented in detail by type and duration of disease. Our review will cover patient categories in the schizophrenia spectrum disorders. These disorders look similar, with psychosis as a common symptom, but there are slight differences in duration and symptoms of the diseases, and finding suitable treatments is difficult considering these differences. For example, most schizophreniform disorders and brief psychotic disorders were thought to have fast remissions and retain this status relatively well [19–21]. In addition, depressive and manic dimensions are significantly higher in schizoaffective patients than in schizophrenia patients [22]. Delusional disorders cause no functional damage other than in the areas of life associated with delusions [19]; thus, general function is evaluated as being higher than that of schizophrenia patients [22]. If we can numerically determine which patients or symptoms each community-based intervention is particularly effective for, it will ultimately help patients, caregivers, and community-service providers identify the most suitable community services.

### Aims

Analyzing the effectiveness of community-based intervention studies developed and applied worldwide to date through a comprehensive systematic review proves a necessary step in developing community-based intervention programs for patients with schizophrenia. This systematic review allows for the planning of community-based interventions in countries where foundations for community-based interventions have not yet been established. Furthermore, new evidence-based recommendations may be provided for countries where existing community intervention has been established. Thus, this review will aim to

- Identify the relapse and remission rates for patients with schizophrenia who have participated in community-based intervention programs.
- Identify the quality of life for patients with schizophrenia and their families who have participated in community-based intervention programs.

## Materials and design

The present protocol has been registered within the PROSPERO database (registration number CRD42019145660) and is being reported in accordance with the reporting guidance provided in the Preferred Reporting Items for Systematic Reviews Meta-Analyses Protocols (PRISMA-P) statement [23] (see checklist in Additional file 1). This systematic review will be conducted in accordance with the Cochrane Handbook for Systematic Reviews of Intervention, 2nd edition [24]. As recommended by the handbook, we derived the review question through consultation with stakeholders, consisting of a community mental health center practitioner, a psychiatric nurse, and mental health policy experts. Finally, the search strategy was reviewed by search experts (medical librarians).

### Review inclusion criteria

#### Participants

This review will include all patients with schizophrenia spectrum disorders who received/were subject to a community-based intervention program. The patients considered in our study are diagnosed as having schizophrenia spectrum disorders as defined by the Diagnostic and Statistical Manual of Mental Disorder, 5th edition (DSM-5) [19]. Schizophrenia spectrum disorders include schizophrenia, schizoaffective disorder, schizophreniform disorder, brief psychotic disorder, delusional disorder, and psychotic disorder not otherwise specified. Because psychotic symptoms are common characteristics, all diagnoses are included in the review literature, but the results are presented separately according to the individual diagnosis.

In addition to the differences inherent to their diagnosis, patients with these diagnoses may experience differences in treatment effects over the duration of the disease, so the results will be presented separately, based on the duration of the patients’ illness (divided into the first episode or chronic status).

#### Intervention

This review will consider studies that evaluate any type of intervention programs that originated from the community-based intervention program for patients with schizophrenia. Those interventions may include but are not limited to, case management, cognitive behavioral therapy, occupational rehabilitation, and physical intervention programs.

#### Comparators

The comparison groups will include one group of patients with schizophrenia spectrum disorders who are receiving the usual care (outpatient treatment that includes only medication) and a group who also receive community-based interventions in addition to the usual care.

### Outcomes

Exploratory analysis will be conducted to identify the relapse, recovery, and/or remission rates of psychotic symptoms. In addition, the review will include the patients’ symptomatic severity and quality of life as outcomes of the community-based intervention. The patients’ conditions will be verified by the number and duration of hospitalizations after the community-based intervention, by their scores on the Positive and Negative Syndrome Scale (PANSS), Brief Psychiatric Rating Scale (BPRS), and Global Assessment of Functioning (GAF). Quality of life will be defined by the patients’ and their respective caregivers’ Quality of Life Scale scores. Thus, any studies that report any of the above outcomes will be included.

### Study design

This review will only consider randomized controlled trials (RCT). Non-randomized controlled trials (non-RCT), cohort studies, case studies, and review articles will be excluded. It will report the specific characteristics of all included studies, using the inclusion criteria that the studies must be written in either English or Korean. We will not include data in the study results because it is difficult to extract data accurately for non-English or non-Korean written papers, but we will inform readers of ‘Studies Awaiting Classification’ through the PRISMA flowchart so that they can be used in other possibly-relevant reports. As community-based mental health services would have been implemented at various times in different countries, we will place no restrictions on the date of publication, and will consider any papers published until 6 January 2021. We will include not only peer reviewed papers but also gray literature (e.g., conference papers, reports, theses/dissertations, protocols) to reduce the bias in our research findings. Therefore, in cases of conference proceedings or protocols without data, we will manually search for full-text or contact the author to request unpublished data for systematic review.

### Electronic bibliographic databases

Electronic searches will be conducted on the following databases from inception to 6 January 2021: MEDLINE (PubMed), Cochrane Central Register of Controlled Trials (CENTRAL), PsycINFO, EMBASE, the Cumulative Index to Nursing and Allied Health Literature (CINAHL), and Research Information Sharing Service (Korean database). To supplement these searches and reduce publication bias, we will expand our search for gray literature using System for Information on Grey Literature in Europe (SIGLE) and GreySource. We will search dissertations and theses by using Open Access Theses and Dissertations (OATD) and ProQuest Dissertations and Theses Global (PQDT). We will manually search the ClinicalTrials.gov website and International Clinical Trials Registry Platform search portal to identify relevant studies. Other avenues for identifying studies will be to use advanced search on Google Scholar, Scopus, and Web of Science. If only conference proceedings or trial protocols are in the search document, we will email the author to request the unreleased data.

### Search strategy

The search strategy aims to find published or unpublished that are in accordance with the Population Intervention Comparison and Outcome Process. An initial search of PubMed will utilize text words related to the systematic review research question: “schizophrenia” and “community based” or “community mental health services.” Then, we will identify relevant keywords by an analysis of the text words contained in the title and abstract, and of the index terms used to describe the relevant articles to refine our search. Draft search strategies are provided in Additional file 2.

### Study screening and selection

Search results will be downloaded using Endnote software, X9 version, and duplicate studies will be eliminated. In the first review, we will review the title and abstract of the selected studies to identify populations, intervention and outcome variables, and study designs to eliminate non-relevant literature; in the second review, a full-text review will identify the final literature of the selected studies. Each selected study will be independently reviewed by two researchers and will be cross-reviewed by both researchers. During the process, if opinions do not agree among researchers, the text will be reviewed together until the researchers reach an agreement.

### Data extraction

Data extraction will include specificities about populations, types of interventions, study designs, and outcome variables. Researchers will select five articles to create a pilot-format data extraction tool, and this tool will use the EPPI reviewer version 4.11.5.2 (http://eppi.ioe.ac.uk/).

In addition to the outcome data, descriptive details such as study designs (e.g., multicenter or cluster), participants’ characteristics (e.g., age, gender, diagnosis, disease status), methods used in the analysis, and methods of intervention (handling) will also be recorded and reviewed. The amount, duration, frequency, and intensity of each reported intervention will also be included in the record. The demographic characteristics and types of interventions will also be specified to enhance the study analysis and synthesis.

If there are any missing or unclear data, we will contact the author of the original research to clarify.

### Assessing risk of bias

For RCT studies, we will use the Cochrane RoB 2.0 tool [25]. RoB is a tool that combines both the checklist method and area evaluation method, and it is an important tool because its area evaluation randomizes sequence generation, which blinds parts of the study and study personnel, blinds the outcome assessments, and does not include incomplete outcome data, which helps us avoid selective reporting and other possible types of biased selection. To avoid the risk of biases in each question, they will be judged as “high,” “low,” and “uncertified” bias, in accordance with the specific presented guidelines. Any disagreement will be resolved by discussion.

## Analysis

### Descriptive analysis

Our review results will be descriptively synthesized and analyzed. The structure of the studies will be described, and they will be structured according to the following characteristics:

- The characteristics of target populations
- The type of intervention
- Intervention exposure (e.g., intervention duration/times, individual or team approach)
- The type of outcome

### Statistical analysis

We will perform a meta-analysis that will first calculate summary estimates of individual studies. In this study, the results will be reported and divided into studies with discrete (hospitalization incidence rate) or continuous (hospitalization period; clinical scale involving PANSS, GAF, BPRS; quality of life) variables. The hospitalization rate will be calculated via odds ratio and other continuous variables (e.g., t, F, p), and the standardized mean difference will be used to calculate effect sizes, which will be calculated by merging the effect sizes of individual studies using the RevMan 5.3 software.

We will quantify statistical heterogeneity by estimating the variance between studies using *I*^2^ statistic [26]. The *I*^2^ is the proportion of variation in study outcomes between studies that is due to genuine variation rather than random error. I^2^ ranges between 0% and 100% (with values of 0–25% and 75–100% taken to indicate low and considerable heterogeneity, respectively). If feasible and appropriate, outcome data will be used to perform random effects meta-analyses because of heterogeneity is expected a priori. The random effects model assumes the study level effect estimates follow a normal distribution, considering both within-study and between-study variation. Factors expected to contribute to heterogeneity include the clinical characteristics of the patients, including the severity of the disease, the comorbidity, the exact diagnosis, and the duration of the disease, which have all been reported to have an effect on treatment in previous studies [27–30]. Therefore, the subgroups will be set up in consideration of clinical characteristics and types of interventions, so that the likelihood of statistical errors is reduced. If quantitative synthesis is not appropriate due to high heterogeneity, we will only perform a descriptive synthesis.

### Confidence in cumulative evidence

For strength of evidence related to all outcomes, we will assess evidence using the Grades of Recommendation Assessment, Development and Evaluation (GRADE) method, and it will be judged as “high,” “moderate,” “low,” and “very low” [31].

## Discussion

This systematic review of the effect of community-based interventions on patients with schizophrenia spectrum disorders will provide a detailed summary of the available evidence on the effectiveness of this type of intervention, and we intend to provide specific guidelines to help improve the outcomes of community mental health services. This will lay the groundwork for its role in enabling community centers to actively support patients diagnosed with the schizophrenia spectrum disorders. However, as this study focuses only on the effectiveness of community-based interventions, it is only discussed in patients who have already enrolled in community centers. In other words, it does not address direct strategies for connecting patients with schizophrenia spectrum disorders from hospital to community or finding untreated patients in the community. If the positive community-based intervention effect is known in detail through this systematic review, although it is expected to have a positive impact on making patient enrollment in the community center easier, follow-up study on effective ways to connect patients and community centers after discharge may also be considered. Findings of this study will be disseminated through publication in a peer-reviewed journal and conference presentations to mental healthcare providers in community center.

Among the final selected studies, it is used to derive effects by synthesizing the results of studies evaluated from risk of bias to ‘low’ or ‘some concerns.’ Since we only consider studies written in English and Korean, publication bias attributed to them may affect the results of the study.

The searched literature will be covered by January 2021, but if the systematic review is delayed due to the unexpected number of studies requiring full text review, the search period can be extended to add the latest literature. When conducting a review, all amendments made, including these further searches, will be outlined in PROSPERO and reported in the final manuscript.

## Supplementary Information

**Additional file 1.** PRISMA-P 2015 Checklist

**Additional file 2.** Search strategy

## Data Availability

The study protocol does not include the dataset, but the search strategy is included.

## Abbreviations

BPRS: Brief Psychiatric Rating Scale
CINAHL: Cumulative Index to Nursing and Allied Health Literature
GAF: Global Assessment of Functioning
GRADE: Grades of Recommendation Assessment, Development and Evaluation
Non-RCT: Non-randomized control trials
PANSS: Positive and Negative Syndrome Scale
PICO: Population Intervention Comparison and Outcome
PRISMA: Preferred Reporting Items for Systematic Reviews and Meta-analyses
QOLS: Quality of Life Scale
RCT: Randomized control trials
SMD: Standardized mean difference
