# Supplementary material for: Effectiveness of community-based interventions for patients with schizophrenia spectrum disorders: a study protocol for a systematic review
Source: Syst Rev. 2021 Apr 13;10:106. doi: 10.1186/s13643-021-01662-0 (PMC8042964; doi:10.1186/s13643-021-01662-0)
Supplement: Supplementary file 2 — Additional file 2. Search strategy [file 13643_2021_1662_MOESM2_ESM.docx]

PsycINFO

Searching date : 06/Jan/2021

| 1 | Schizophrenia | 146,565 |
| --- | --- | --- |
| 2 | Schizophrenia Spectrum and Other Psychotic Disorders | 1034 |
| 3 | Schizophreni* OR Schizophrenic Disorder* OR Schizophrenia Spectrum | 150,949 |
| 4 | psychosis* OR psychotic* OR delusion* | 127,830 |
| 5 | #1 OR #2 OR #3 OR #4 | 200,766 |
| 6 | "Community Mental Health Service*" | 14,841 |
| 7 | "Community Mental Health Center*" | 5,421 |
| 8 | "Community based" | 31,539 |
| 9 | intervention* OR program* OR management* OR treatment* | 1,801,081 |
| 10 | #8 AND #9 | 23,105 |
| 11 | #6 OR #7 OR #10 | 39,688 |
| 12 | "Patient Readmission" | 2,068 |
| 13 | Recurrence | 16,886 |
| 14 | "symptom relapse*" OR Relapse* OR Recrudescenc* OR "Hospital Readmission*" | 29,427 |
| 15 | #12 OR #13 OR #14 | 42,217 |
| 16 | "Quality of Life" | 89,893 |
| 17 | "EQ 5D*" OR EuroQoL* OR QoL* | 15,366 |
| 18 | #16 OR #17 | 91,850 |
| 19 | #15 OR #18 | 131,925 |
| 20 | #5 AND #11 AND #19 | 631 |
| Search strategy | (Schizophrenia OR (Schizophrenia Spectrum and Other Psychotic Disorders) OR (Schizophreni* OR Schizophrenic Disorder* OR Schizophrenia Spectrum) OR (psychosis* OR psychotic* OR delusion*)) AND ("Community Mental Health Service*" OR "Community Mental Health Center*" OR ("Community based" AND (intervention* OR program* OR management* OR treatment*))) AND (("Quality of Life" OR ("EQ 5D*" OR EuroQoL* OR QoL*)) OR ("Patient Readmission" OR Recurrence OR ("symptom relapse*" OR Relapse* OR Recrudescenc* OR "Hospital Readmission*"))) | 631 |

| PubMed  Searching date : 06/Jan/2021 |
| --- |

| 1 | "Schizophrenia"[Mesh] | 147,178 |
| --- | --- | --- |
| 2 | "Schizophrenia Spectrum and Other Psychotic Disorders"[Mesh] | 149,594 |
| 3 | "Hallucinations"[Mesh] | 18,712 |
| 4 | "Delusions"[Mesh] | 12,573 |
| 5 | "psychotic disoders"[Mesh] | 66,237 |
| 6 | Schizophreni*[TIAB] OR Schizophrenic Disorder*[TIAB] OR Schizophrenia Spectrum[TIAB] OR Psychotic*[TIAB] | 151,245 |
| 7 | #1 OR #2 OR #3 OR #5 OR #6 OR #7 | 216,196 |
| 8 | "Community Mental Health Services"[Mesh] | 40,102 |
| 9 | "Community Mental Health Centers"[Mesh] | 6,780 |
| 10 | Community based[TIAB] | 61,813 |
| 11 | intervention*[TIAB] OR program*[TIAB] OR management*[TIAB] OR treatment*[TIAB] | 6,644,498 |
| 12 | #10 AND #11 | 35,890 |
| 13 | #8 OR #9 OR #12 | 75,997 |
| 14 | "Patient Readmission"[Mesh] | 25,459 |
| 15 | "Recurrence"[Mesh] | 704,286 |
| 16 | symptom relapse*[TIAB] OR Relapse*[TIAB] OR Recrudescenc*[TIAB] OR Hospital Readmission*[TIAB] | 176,063 |
| 17 | #14 OR #15 OR #16 | 838,221 |
| 18 | "Quality of Life"[Mesh] | 420,086 |
| 19 | EQ 5D*[TIAB] OR EuroQoL*[TIAB] | 11,530 |
| 20 | Quality of life[TIAB] OR QoL[TIAB] | 287,688 |
| 21 | #18 OR #19 OR #20 | 423,189 |
| 22 | #17 OR #21 | 1,236,279 |
| 23 | #7 AND #13 AND #22 | 911 |
| Search strategy | ("Schizophrenia"[MeSH Terms] OR "Schizophrenia"[All Fields] OR "schizophrenias"[All Fields] OR "schizophrenia s"[All Fields] OR ("schizophrenia spectrum and other psychotic disorders"[MeSH Terms] OR ("Schizophrenia"[All Fields] AND "Spectrum"[All Fields] AND "other"[All Fields] AND "psychotic"[All Fields] AND "disorders"[All Fields]) OR "schizophrenia spectrum and other psychotic disorders"[All Fields]) OR ("hallucinations"[MeSH Terms] OR "hallucinations"[All Fields] OR "hallucinate"[All Fields] OR "hallucinated"[All Fields] OR "hallucinating"[All Fields] OR "hallucination"[All Fields] OR "hallucinative"[All Fields] OR "hallucinator"[All Fields] OR "hallucinators"[All Fields]) OR ("psychotic disorders"[MeSH Terms] OR ("psychotic"[All Fields] AND "disorders"[All Fields]) OR "psychotic disorders"[All Fields]) OR ("schizophreni*"[Title/Abstract] OR "schizophrenic disorder*"[Title/Abstract] OR "schizophrenia spectrum"[Title/Abstract] OR "psychotic*"[Title/Abstract]) OR ("delusions"[MeSH Terms] OR "delusions"[All Fields] OR "delusion"[All Fields] OR "delusive"[All Fields])) AND ("community mental health services"[MeSH Terms] OR ("Community"[All Fields] AND "mental"[All Fields] AND "health"[All Fields] AND "services"[All Fields]) OR "community mental health services"[All Fields] OR ("community mental health centers"[MeSH Terms] OR ("Community"[All Fields] AND "mental"[All Fields] AND "health"[All Fields] AND "centers"[All Fields]) OR "community mental health centers"[All Fields]) OR (("intervention*"[Title/Abstract] OR "program*"[Title/Abstract] OR "management*"[Title/Abstract] OR "treatment*"[Title/Abstract]) AND "community based"[Title/Abstract])) AND ("patient readmission"[MeSH Terms] OR ("patient"[All Fields] AND "readmission"[All Fields]) OR "patient readmission"[All Fields] OR ("recurrance"[All Fields] OR "recurrence"[MeSH Terms] OR "recurrence"[All Fields] OR "recurrences"[All Fields] OR "recurrencies"[All Fields] OR "recurrency"[All Fields] OR "recurrent"[All Fields] OR "recurrently"[All Fields] OR "recurrents"[All Fields]) OR ("symptom relapse*"[Title/Abstract] OR "relapse*"[Title/Abstract] OR "recrudescenc*"[Title/Abstract] OR "hospital readmission*"[Title/Abstract]) OR ("quality of life"[MeSH Terms] OR ("Quality"[All Fields] AND "life"[All Fields]) OR "quality of life"[All Fields] OR ("eq 5d*"[Title/Abstract] OR "euroqol*"[Title/Abstract]) OR ("quality of life"[Title/Abstract] OR "QoL"[Title/Abstract]))) | 911 |

Embase

Searching date : 06/Jan/2021

| 1 | exp schizophrenia spectrum disorder/ or exp schizophrenia/ | 186665 |
| --- | --- | --- |
| 2 | exp psychosis/ | 284782 |
| 3 | schizophrenia.ab,kw,ti. | 157557 |
| 4 | schizophrenia spectrum disorder.ab,kw,ti. | 1158 |
| 5 | psychosis.ab,kw,ti. | 61958 |
| 6 | 1 or 2 or 3 or 4 or 5 | 310576 |
| 7 | exp community mental health center/ or exp community mental health service/ | 3350 |
| 8 | community based.ab,kw,ti. | 80821 |
| 9 | ('intervention*' or 'program*' or 'management*' or 'treatment*').ti,ab,kw. | 8992968 |
| 10 | 8 and 9 | 47776 |
| 11 | 7 or 10 | 50958 |
| 12 | exp hospital readmission/ | 69273 |
| 13 | exp recurrent disease/dm, rh [Disease Management, Rehabilitation] | 445 |
| 14 | ('symptom relapse*' or 'Relapse*' or 'Recrudescenc*' or 'Hospital Readmission*').ti,ab,kw. | 303457 |
| 15 | 12 or 13 or 14 | 362021 |
| 16 | exp "quality of life"/ or exp "quality of life assessment"/ or exp "quality of life index"/ | 550391 |
| 17 | ('QoL*' or 'Quality of life*' or 'EQ 5D*' or 'EuroQol*').ti,ab,kw. | 487701 |
| 18 | 16 or 17 | 655907 |
| 19 | 15 or 18 | 1001866 |
| 20 | 6 and 11 and 19 | 295 |

CINAHL

Searching date : 06/Jan/2021

| 1 | (MH "Schizophrenia+") | 26,721 |
| --- | --- | --- |
| 2 | Schizophreni* OR Schizophrenic Disorder* OR Schizophrenia Spectrum | 34,725 |
| 3 | psychosis* OR psychotic* OR delusion* | 26213 |
| 4 | #1 OR #2 OR #3 | 52,282 |
| 5 | (MH "Community Mental Health Services+") OR (MH "Mental Health") OR (MH "Mental Health Services+") | 119,680 |
| 6 | Community based | 40,920 |
| 7 | intervention* OR program* OR management* OR treatment* | 2,147,963 |
| 8 | #6 AND #7 | 25,040 |
| 9 | #5 OR #8 | 142,546 |
| 10 | (MH "Readmission") OR (MH "Recurrence") | 63,560 |
| 11 | symptom relapse* OR Relapse* OR Recrudescenc* OR Hospital Readmission* | 49,507 |
| 12 | #10 OR #11 | 88,793 |
| 13 | (MH "Quality of Life") | 115,918 |
| 14 | EQ 5D* OR EuroQoL* OR QoL* | 18,573 |
| 15 | #13 OR #14 | 119,923 |
| 16 | #12 OR #15 | 206,240 |
| 17 | #4 AND #9 AND #16 | 410 |

Cochrane

Searching date : 06/Jan/2021

| 1 | MeSH descriptor: [Schizophrenia Spectrum and Other Psychotic Disorders] explode all trees | 9199 |
| --- | --- | --- |
| 2 | schizo*':ab,ti,kw OR 'psychosis*':ab,ti,kw OR 'schizophrenia spectrum*':ab,ti,kw | 21337 |
| 3 | #1 OR #2 | 22031 |
| 4 | MeSH descriptor: [Mental Health Services] explode all trees | 6824 |
| 5 | MeSH descriptor: [Community Mental Health Centers] explode all trees | 117 |
| 6 | 'community based':ti,ab,kw | 23125 |
| 7 | 'intervention*' OR 'program*' OR 'management*' OR 'treatment*':ti,ab,kw | 1070201 |
| 8 | #6 AND 7 | 21061 |
| 9 | #4 OR #5 OR #8 | 27035 |
| 10 | MeSH descriptor: [Patient Readmission] explode all trees | 1071 |
| 11 | MeSH descriptor: [Recurrence] explode all trees | 12158 |
| 12 | symptom relapse*' OR 'Relapse*' OR 'Recrudescenc*' OR 'Hospital Readmission*':ti,ab,kw | 44966 |
| 13 | #10 OR #11 OR #12 | 53926 |
| 14 | MeSH descriptor: [Quality of Life] explode all trees | 24223 |
| 15 | QoL*':ab,ti,kw OR 'Quality of life*':ab,ti,kw OR 'EQ 5D*':ab,ti,kw OR 'EuroQol*':ab,ti,kw | 124529 |
| 16 | #14 OR #15 | 124529 |
| 17 | #3 AND #9 AND #17 | 438 |

RISS (Korean Database)

Searching date : 06/Jan/2021

| Search | Query | Results |
| --- | --- | --- |
| #1 | "Schizophrenia" \| Schizophrenic Disorder \| Schizophrenia Spectrum \| psychotic \| psychosis \| "조현병" | 848 |
| #2 | "Community Mental Health Services" \| Community based intervention \| Community based program \| Community based management \| Community based Treatment \| 지역사회중재 \| 개입 \| 치료 \| 프로그램 | 122,521 |
| #3 | "Patient Readmission" \| "Recurrence" \| "symptom relapses" \| Relapse \| Recrudescence \| Hospital Readmission \| 재입원 \| 재발 \| "Quality of Life" \| "Qol" \| "EQ 5D" \| "삶의 질" | 25205 |
| #4 | #1 AND #2 AND #3 | 34 |

| Korean | English |
| --- | --- |
| 조현병 | Schizophrenia |
| 지역사회중재 | Community based intervention |
| 개입 | Intervention |
| 치료 | Treatment |
| 프로그램 | Program |
| 재입원 | Hospital Readmission |
| 재발 | Relapse |
| 삶의 질 | Quality of life |
